# Forecasting and optimizing *Agrobacterium*-mediated genetic transformation via ensemble model- fruit fly optimization algorithm: A data mining approach using chrysanthemum databases

**DOI:** 10.1371/journal.pone.0239901

**Published:** 2020-09-30

**Authors:** Mohsen Hesami, Milad Alizadeh, Roohangiz Naderi, Masoud Tohidfar

**Affiliations:** 1 Department of Plant Agriculture, Gosling Research Institute for Plant Preservation, University of Guelph, Guelph, ON, Canada; 2 Department of Botany, University of British Columbia, Vancouver, BC, Canada; 3 Department of Horticultural Science, Faculty of Agriculture, University of Tehran, Karaj, Iran; 4 Department of Plant Biotechnology, Faculty of Sciences & Biotechnology, Shahid Beheshti University, G.C., Tehran, Iran; Lovely Professional University, INDIA

## Abstract

Optimizing the gene transformation factors can be considered as the first and foremost step in successful genetic engineering and genome editing studies. However, it is usually difficult to achieve an optimized gene transformation protocol due to the cost and time-consuming as well as the complexity of this process. Therefore, it is necessary to use a novel computational approach such as machine learning models for analyzing gene transformation data. In the current study, three individual machine learning models including Multi-Layer Perceptron (MLP), Adaptive Neuro-Fuzzy Inference System (ANFIS), and Radial Basis Function (RBF) were developed for forecasting *Agrobacterium*-mediated gene transformation in chrysanthemum based on eleven input variables including *Agrobacterium* strain, optical density (OD), co-culture period (CCP), and different antibiotics including kanamycin (K), vancomycin (VA), cefotaxime (CF), hygromycin (H), carbenicillin (CA), geneticin (G), ticarcillin (TI), and paromomycin (P). Consequently, best-obtained results were used in the fusion process by bagging method. Results showed that ensemble model with the highest R^2^ (0.83) had superb performance in comparison with all other individual models (MLP:063, RBF:0.69, and ANFIS: 0.74) in the validation set. Also, ensemble model was linked to Fruit fly optimization algorithm (FOA) for optimizing gene transformation, and the results showed that the maximum gene transformation efficiency (37.54%) can be achieved from EHA105 strain with 0.9 OD_600_, for 3.8 days CCP, 46.43 mg/l P, 9.54 mg/l K, 18.62 mg/l H, and 4.79 mg/l G as selection antibiotics and 109.74 μg/ml VA, 287.63 μg/ml CF, 334.07 μg/ml CA and 87.36 μg/ml TI as antibiotics in the selection medium. Moreover, sensitivity analysis demonstrated that input variables have a different degree of importance in gene transformation system in the order of Agrobacterium strain > CCP > K > CF > VA > P > OD > CA > H > TI > G. Generally, the developed hybrid model in this study (ensemble model-FOA) can be employed as an accurate and reliable approach in future genetic engineering and genome editing studies.

## Introduction

Horticulture plants including fruits, vegetables, grapes, and ornamental plants are raw material and used by people for food, either as edible products or for culinary ingredients, for medicinal use or ornamental and aesthetic purposes. They are a genetically very diverse group and play a major role in modern society and the economy [1–4]. Chrysanthemum (*Dendranthema × grandiflorum*) can be categorized as the second most economically important ornamental species due to its color and morphological diversity [5]. Moreover, chrysanthemum has been used as a model plant for color modification [6]. Conventional propagation and breeding approaches are not able to meet the increasing demands of the market for this valuable ornamental plant. Therefore, novel biotechnological methods such as genetic manipulation and gene editing such as CRISPR/Cas9 can be employed in order to satisfy the demands of consumers. Optimizing the gene transformation protocol can be considered as the first and foremost step in successful genetic engineering and gene editing studies [6, 7]. Many factors such as *in vitro* regeneration parameters (temperature, type and age of explant, quality and intensity of light, type and concentration of plant growth regulators, medium compositions), bacterial optical cell density, antibiotic and chemical stimulants concentrations, and inoculation duration (immersion time), play an important role in the efficiency of gene transformation [5]. Establishing an optimized protocol for genetic *Agrobacterium*-mediated transformation can be considered as a highly complex system, and it is critical to comprehend the effect of different factors prompting the T-DNA delivery into various explants [5, 8]. Subsequently, further analyses are essential to check T-DNA integration and stability and to achieve the efficiency parameter of gene transformation [9]. However, it is usually difficult to achieve an optimized gene transformation protocol due to the cost and time-consuming as well as the complexity of this process. Therefore, gene transformation can be considered as a multi-variable and non-linear biological process. Hence, conventional linear computational methods such as simple regression are not appropriate for analyzing biological systems such as gene transformation. Machine learning algorithms as a non-linear approach can be considered as a suitable computational methodology for predicting and optimizing different complex biological systems. Several studies have proved the usefulness of ANN for modeling and predicting in vitro culture processes such as in vitro secondary metabolite production, shoot proliferation and somatic embryogenesis [10–16]. Nowadays, the necessity of increased precision and accuracy of machine learning algorithms has encouraged researchers to develop applicable methods such as ensemble approaches. The key idea of ensemble is fusing or combining data derived from fused information in order to provide more precise estimations in comparing with using individual model [17]. Many researchers in several fields of study have used ensemble models [18–20]. At more complex features such as gene transformation, ensemble methods could be used to integrate the advantages and strengths of individual models. Several studies have demonstrated that ensemble models can be more reliable and accurate to model complex systems [17–20]. Therefore, ensemble model can be considered as a reliable tool to help the handling of complex systems and to data mining. Data mining can be defined as the process of discovering and understanding previously unknown relationships and dependencies in datasets. In fact, data mining can be applied to generate and model rules able to enhance knowledge or further insight from experimental data [21].

However, difficulty in achieving an optimized solution can be considered as one of the demerit points of most machine learning algorithms [22–29]. To overcome this bottleneck, Zhang *et al*. [30] employed the genetic algorithm (GA) as one of the common optimization algorithms for optimizing relative humidity, light duration, agar concentration, and culture temperature in order to maximize indirect shoot organogenesis in *Cucumis melo*. In another study, Non-dominated Sorting Genetic Algorithm-II (NSGA-II) was employed to optimize different types and concentrations of disinfectants as well as immersion time for maximizing explant viability and minimizing in vitro contamination in chrysanthemum [10]. However, most studies have found the optimized solution by trials and error [14, 31–36]. Fruit fly optimization algorithm (FOA) suggested by Pan [37] is a new evolutionary optimization and computation approach. This novel optimization algorithm has the merits of being simple to comprehend and to be written into linguistic terms which is not too complex compared with other optimization algorithms [38]. Therefore, this study has attempted to apply the FOA to find the optimal levels of different factors involved in gene transformation.

In the current study, data mining by using ensemble strategy was employed to assess the effect and importance of different factors in *Agrobacterium*-mediated genetic transformation.

Data dispersed into several single chrysanthemum databases was assembled in order to model them and obtain further insight into the effect of different factors involved in chrysanthemum gene transformation. Furthermore, FOA was linked to the ensemble model to find the optimal level of factors involved in chrysanthemum gene transformation. According to the best of our knowledge, this study is the first report of the application of ensemble model in the field of genetic engineering.

## Results

### Evaluating and comparing different individual (MLP, RBF, and ANFIS) models and ensemble method

Three individual models including MLP, RBF, and ANFIS were applied for forecasting gene transformation efficiency in chrysanthemum based on eleven inputs including *Agrobacterium* strain, optical density (OD), co-culture period (CCP), and different antibiotics including kanamycin (K), vancomycin (VA), cefotaxime (CF), hygromycin (H), carbenicillin (CA), geneticin (G), ticarcillin (TI), and paromomycin (P). In order to improve forecasting results, the best estimations obtained by three individual models were fused through the bagging method.

**Fig 1:**
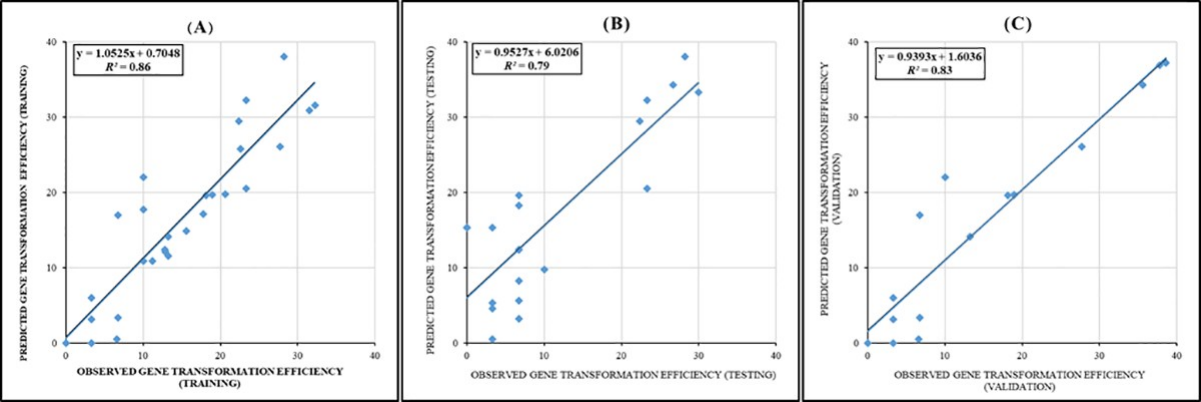
Scatter plot of model predicted vs. observed data of chrysanthemum gene transformation efficiency by ensemble model. (A) Training set, (B) Testing set, and (C) Validation set.

The efficiency of the individual and ensemble models was determined based on the assessment of forecasted and observed data. All the R^2^ of testing, training, and validation datasets were over 63%, 69%, and 73% for MLP, RBF, and ANFIS models, respectively (Table 1). According to Table 1, the ensemble model had the better predictive ability on forecasting gene transformation efficiency (R^2^ > 0.86, 079, and 0.83 for training, testing and validation sets, respectively) compared with individual models. The good fit of the ensemble model can be traced by the correlation between observed and forecasted data for gene transformation efficiency (Fig 1). Also, RMSE and MBE, same as R^2^, in ensemble model were better than individual models (Table 1). Based on the performance criteria that was mentioned in Table 1, ensemble model was able to efficiently explain the performances of *Agrobacterium*-mediated gene transformation to different studied factors.

**Table 1:** Performance criteria of individual and ensemble models for gene transformation efficiency of chrysanthemum in training, testing, and validation processes.

Model	*R*^*2*^			RMSE			MBE		
	Training	Testing	Validation	Training	Testing	Validation	Training	Testing	Validation
MLP	0.71	0.68	0.63	1.24	2.63	2.87	0.43	0.66	-0.84
RBF	0.73	0.71	0.69	1.21	1.76	1.96	-0.37	0.69	1.07
ANFIS	0.77	0.73	0.74	0.91	1.05	1.01	0.32	-0.54	-0.96
Ensemble	0.86	0.79	0.83	0.93	0.83	0.88	0.26	0.19	0.21

R^2^: coefficient of determination; MBE: Mean Bias Error; RMSE: Root Mean Square Error; MLP: Multi-Layer Perceptron; ANFIS: Adaptive Neuro-Fuzzy Inference System; RBF: Radial Basis Function.

### Optimizing gene transformation through FOA

The aim of the current study not only was to forecast the gene transformation but also was to find an optimized level of *Agrobacterium* strain, OD, CCP, and different antibiotics including K, VA, CF, H, CA, G, TI, and P for the maximum *Agrobacterium*-mediated gene transformation efficiency in chrysanthemum. FOA was linked to ensemble model for achieving the optimal level of factors involved in gene transformation. The result of the optimization process was summarized in Table 2. According to Table 2, the maximum gene transformation efficiency (37.54%) can be achieved from EHA105 strain with 0.9 OD_600_, for 3.8 days CCP, 46.43 mg/l P, 9.54 mg/l K, 18.62 mg/l H, and 4.79 mg/l G as selection antibiotics and 109.74 μg/ml VA, 287.63 μg/ml CF, 334.07 μg/ml CA and 87.36 μg/ml TI as antibiotics in the selection medium.

**Table 2:** The results of optimization process via FOA for gene transformation efficiency of chrysanthemum.

Input											Gene transformation efficiency (%)
Agrobacterium Strain	OD	CCP	Antibiotics for selecting transgenic tissue (mg/l)				Antibiotics (μg/ml)				
			K	H	P	G	VA	CF	CA	TI	
EHA105	0.9 (660)	3.8	9.54	18.62	46.43	4.79	109.74	287.63	334.07	87.36	37.54

OD: Optical density; CCP: co-culture period; K: kanamycin; VA: vancomycin; CF: cefotaxime; H: hygromycin; CA: carbenicillin; G: geneticin; TI: ticarcillin; P: paromomycin.

### Sensitivity analysis of the models

Databases were also used to determine the overall VSR for identifying the comparative rank of inputs. The results of sensitivity analysis were presented in Table 3. Based on sensitivity analysis, *Agrobacterium*-mediated gene transformation was more sensitive to *Agrobacterium* strain, followed by CCP, K, CF, VA, P, OD, CA, H, TI, and G.

**Table 3:** The results of sensitivity analysis on the developed ensemble model to rank the importance of factors involved in *Agrobacterium*-mediated gene transformation of chrysanthemums using *GUS* gene.

Item	Agrobacterium Strain	OD	CCP	K	H	P	G	VA	CF	CA	TI
VSR	1.86	1.06	1.73	1.54	0.91	1.025	0.87	1.23	1.47	0.94	0.88
Rank	1	7	2	3	9	6	11	5	4	8	10

OD: Optical density; CCP: co-culture period; K: kanamycin; VA: vancomycin; CF: cefotaxime; H: hygromycin; CA: carbenicillin; G: geneticin; TI: ticarcillin; P: paromomycin; VSR: variable sensitivity ratio.

## Discussion

The *Agrobacterium*-mediated gene transformation of the chrysanthemum was widely studied by discovering the susceptibility of different chrysanthemum cultivars to *Agrobacterium tumefaciens* [5, 9]. However, several studies have reported some obstacles to establish and develop chrysanthemum gene transformation system such as chimeric plant regeneration consisting of both non-transgenic and transgenic tissues [39, 40], low efficiency of gene transformation [41–43], and transgene inactivation [44]. Due to these difficulties and also the complex nature of the gene transformation system, there is a dire need to employ new computational methods to optimize this system. AI models can be considered as a reliable strategy to develop and optimize gene transformation protocols. Although there are no reports to use AI models in genetic engineering and genome editing, several studies have previously proved the reliability and accuracy of AI methodology to predict and optimize different *in vitro* culture processes such as *in vitro* sterilization [45, 46], callogenesis [34, 47, 48], cell growth and protoplast culture [49, 50], somatic embryogenesis [34, 51, 52], shoot regeneration [12, 53–55], androgenesis [33], hairy root culture [56, 57], and rhizogenesis [58]. In the current study, MLP, RBF, ANFIS, and ensemble models, for the first time, were used to develop a suitable model for chrysanthemum gene transformation and compare their prediction accuracy. According to our results, ensemble model had more accuracy than individual models for modeling and predicting the system. Although there is no report regarding the application of AI models in gene transformation studies, in line with our results, comparative studies in other fields revealed the better performance of ensemble models in comparison to individual models [17–20]. On the other hand, one of the weaknesses of using AI models is that it is hard to obtain an optimized solution [10]. To tackle this problem, several studies [10, 11, 13, 45, 54] used GA and NSGA-II to optimize in vitro culture conditions. In the current study, FOA was linked to ensemble model for the optimization process. Based on our results, a hybrid ensemble model and FOA can be considered as an efficient computational methodology for predicting and optimizing *Agrobacterium*-mediated gene transformation.

Agrobacterium strains play a pivotal role in gene transformation [8]. Several studies showed that successfulness in chrysanthemum gene transformation directly depends on selecting a suitable strain [5, 9]. Ledger *et al*. [59] first tried to produce transgenic chrysanthemum through LBA4404, however, low transformation efficiency (1.7%) was observed. Just two years later, Renou *et al*. [42] reported that higher transformation frequency between 5% and 40% can be achieved by using EHA101. Further studies [60, 61] employed LBA4404 and EHA101 to compare the performance of these two strains on the chrysanthemum gene transformation. These studies [60, 61] showed that EHA101 caused to 8.8% gene transformation frequency whereas LBA4404 resulted in 5.2%. Afterward, the efficiency of EHA101 and EHA105 was studied and showed that EHA105 had better performance than EHA101 for chrysanthemum gene transformation [9]. In line with previous studies, our results elucidated that EHA105 is the best strain to obtain the maximum gene transformation frequency.

The selection marker is another factor that plays an important role in gene transformation systems [8]. Due to the fact that in the first study of chrysanthemum gene transformation [62], the neomycin phosphotransferase II (*npt*II) gene was applied as a selection marker, kanamycin has been the main selection antibiotic of transgenic chrysanthemums. However, a high level of kanamycin in the selection medium represses organogenesis due to the sensitivity of chrysanthemum to kanamycin [9]. Other antibiotics, such as geneticin, paromomycin, and hygromycin, have been successfully employed for the detection of transgenic cells of chrysanthemums [42, 61, 63]. Our results showed that the combination of 46.43 mg/l paromomycin, 9.54 mg/l kanamycin, 18.62 mg/l hygromycin, and 4.79 mg/l geneticin is the best antibiotics combination for the selection of transgenic tissues. In accordance with our results, Aida *et al*. [63] reported that paromomycin has less toxic to cells than other antibiotics such as kanamycin, and it can reduce the chance of non-transgenic chrysanthemums escapes. Also, our results showed that cefotaxime can be considered as the best antibiotic for the selection medium. Previous studies [42, 61, 63] have proved the usefulness of cefotaxime in the selection medium.

One of the most important factors in *Agrobacterium*-mediated gene transformation systems is the density of the Agrobacterium strain [5, 9]. Therefore, Optimizing the optimal bacterial inoculation density is very critical because, with higher OD levels, explants are completely colonized by Agrobacterium and, subsequently, bacteria elimination becomes more difficult [8]. Similar to the previous studies [60, 64, 65], our results indicated that transformation efficiency can be improved when an optical density (OD600) of 0.9 would be used. The co-cultivation period is expected to be another important factor in gene transformation and transgenic plant regeneration [8]. According to previous studies [9, 66, 67], the regeneration of chrysanthemum explants following cocultivation with *A*. *tumefaciens* was significantly decreased even when explants were cultured on optimized media. This negative impact was observed when a c-cultivation period of 8d was employed. According to our results, 3.8 days of co-cultivation is the best period for the gene transformation in the chrysanthemum. Similar results have been reported by Teixeira da Silva and Fukai [67] and Shinoyama *et al*. [9].

## Conclusion

Recently, different individual AI models have been widely applied for modeling and predicting *in vitro* culture processes. In the current study, ensemble model for the first time was applied to model and predict gene transformation efficiency and to compare its accuracy with individual models. Our results showed that the ensemble model has better accuracy than MLP, RBF, and ANFIS for modeling and predicting complex systems such as *Agrobacterium*-mediated gene transformation. Also, FOA was able to accurately optimize the chrysanthemum's gene transformation. The results of the current study demonstrate that the developed hybrid model (Ensemble-FOA) can open a reliable and accurate window to a comprehensive study of the plant's biological processes.

## Materials and methods

### Case study and data collection

Several experimental databases were selected from previous studies where detailed descriptions of materials and methods are available [9, 39–44, 59–100]. Data supporting the effect of *Agrobacterium* strain, optical density (OD), co-culture period (CCP), and different antibiotics including kanamycin (K), vancomycin (VA), cefotaxime (CF), hygromycin (H), carbenicillin (CA), geneticin (G), ticarcillin (TI), and paromomycin (P) on gene transformation efficiency of chrysanthemum using *GUS* gene were summarized in Table 4.

**Table 4:** Studies on *Agrobacterium*-mediated gene transformation of chrysanthemums using *GUS* gene.

Input											Gene transformation efficiency (%)	Reference
Agrobacterium strain(s)	OD	CCP	Antibiotics for selecting transgenic tissue (mg/l)				Antibiotics (μg/ml)					
			K	H	P	G	VA	CF	CA	TI		
LBA4404	1.5 (550)	8	-	-	-	-	400	250	-	-	4.3–13.4	Jong *et al*. [68]
LBA4404	0.8 (550)	4	25	-	-	-	100–300	-	-	-	0–4.6	Lemieux *et al*. [62]
LBA4404, A2002	0.1 (660)	2	25	-	-	-	-	-	-	500	0–0.8	Ledger *et al*. [59]
LBA4404, A281, Ach5, C58	0.5 (660)	6	50	-	-	-	400	250	-	-	0–0.75	van Wordragen *et al*. [69]
EHA101	0.1 (660)	3	35	-	-	-	-	250	-	-	0.06	Aida *et al*. [70]
LBA4404, A281, Ach5	0.5 (660)	2	50	-	-	-	400	250	-	-	0–10	Van Wordragen *et al*. [71]
LBA4404	0.6 (660)	2	-	-	-	-	400	250	-	-	1.4–4.6	de Jong *et al*. [66]
LBA4404	0.1 (660)	4	-	-	-	-	-	-	500	-	0–0.4	Courtney-Gutterson *et al*. [72]
EHA101, Ach5, C58, Bo542	0.7 (660)	1	25	5	-	-	400	500	-	-	1.04–12.14	Renou *et al*. [42]
LBA4404, C58	0.5 (660)	2	15–25	-	-	-	-	500	-	-	0–6.3	Lowe *et al*. [73]
A281	0.5 (660)	3	50–100	-	-	-	200	125	-	-	0–2.5	van Wordragen *et al*. [74]
LBA4404	0.1 (660)	3–5	100	-	-	-	-	-	500	-	0–0.4	Courtney-Gutterson *et al*. [75]
B6S3	0.1 (660)	1	100	-	-	-	-	200	-	500	17–47	Pavingerová *et al*. [39]
LBA4404,AGL0	0.4–0.8 (550)	2	10–25	-	-	-	400	250	-	-	0.3–4.3	de Jong *et al*. [41]
EHA105,Ach5,A281,Chry5	2.2 (660)	3–5	50	-	-	-	-	-	500	-	4–7	Urban *et al*. [43]
B6S3	0.1 (660)	1	100	-	-	-	-	200	-	500	3.8–4.7	Benetka and Pavingerová [40]
AGL0	0.5 (540)	2	10	-	-	-	500	250	-	-	0–39.45	de Jong *et al*. [76]
C58,A281	0.1 (660)	2	25	-	-	-	-	500	-	-	0–11.3	Dolgov *et al*. [77]
AGL0	0.7–1 (540)	2	10	-	-	-	400	250	-	-	5.6–15.6	Fukai *et al*. [64]
LBA4404	0.5 (540)	2	50	-	-	-	-	100	-	-	6.9–8.3	Oka *et al*. [78]
A281,GV3101,C58,CBE21	0.6–0.9 (600)	3	10–50	10–15	-	-	-	500	-	-	0–3	Dolgov *et al*. [79]
LBA4404	0.1 (660)	4	20	-	-	-	-	-	-	500	3.4	Boase *et al*. [80]
LBA4404,EHA105 + 2xMOG	0.1 (660)	4	25	-	-	-	-	-	-	500	0–14.2	Boase *et al*. [81]
LBA4404	0.5 (600)	4	25	-	-	-	-	500	-	-	3.4–8.5	Fu *et al*. [82]
LBA4404	0.5 (600)	2	20	-	-	-	-	250	-	-	6.9	Kim *et al*. [83]
LBA4404	0.5 (600)	2	50	-	-	-	-	250	-	-	7.6	Kim *et al*. [84]
EHA105	2.2 (600)	5	-	-	50	-	-	-	500	-	0.5–4.1	John *et al*. [85]
EHA101	0.2 (600)	3	15	15	-	15	-	250	-	-	3.4	Shinoyama *et al*. [86]
LBA4404	0.5 (660)	3	15	15	-	15	-	250	-	-	0–2.5	Takatsu *et al*. [87]
LBA4404	0.1 (660)	3	20	-	-	-	-	250	-	-	1.3–3.1	Young *et al*. [88]
LBA4404	0.5 (600)	2	25	-	-	-	-	250	-	-	6.4%	Shao *et al*. [89]
C58,MP90	0.5 (600)	2	50	-	-	-	-	250	-	-	1.12–1.91	Takatsu *et al*. [44]
EHA101	0.2 (600)	3	-	-	-	20–30	-	250	-	-	3.4	Shinoyama *et al*. [60]
EHA101	0.5 (600)	3	-	10–40	-	-	-	-	500	-	0–2.5	Shirasawa *et al*. [90]
EHA101	1.8 (660)	2	100	-	-	-	125	500	-	-	0–2.3	Tosca *et al*. [91]
AGL0	0.7–1 (540)	2	25	-	-	-	-	125	-	100	0–6.8	Annadana *et al*. [65]
EHA105	2 (600)	2	50	-	-	-	-	-	500	-	3.4–11.4	Zhi-Liang *et al*. [92]
LBA4404,AGL0	0.2 (600)	3	12.5	-	-	-	-	250	-	-	0.5–4.7	Ishida *et al*. [93]
LBA4404	0.5 (600)	3	50	-	-	-	-	500	-	-	1.2–9.4	Jeong *et al*. [94]
EHA101,LBA4404,AGL0	0.1 (600)	4	50	-	-	-	-	-	-	200	3.4–5.9	Kudo *et al*. [95]
LBA4404	0.1 (600)	2	-	-	-	20	-	250	-	-	0–23.9	Shinoyama *et al*. [61]
LBA4404,AGL0	0.6 (550)	3–4	30	-	-	-	-	500	-	-	0–25	Teixeira da Silva and Fukai [67]
LBA4404,AGL0	0.1 (600)		12.5	-	-	-	-	250	-	-	27–38	Toguri *et al*. [96]
AGL0	0.7–1 (540)	4	10	-	-	-	400	250	-	-	31–39	Petty *et al*. [97]
AGL0	0.8 (550)	6	25	-	-	-	500	250	-	-	4.7–13.4	Outchkourov *et al*. [98]
EHA105, AGL0	0.1 (660)	8	-	-	50	-	-	250	-	-	0.5–6.5	Aida *et al*. [63]
EHA105	0.1 (660)	5	-	-	50	-	-	250	-	-	0.5–6.8	Aida *et al*. [99]
EHA105	0.1 (660)	4	-	-	50	-	-	250	-	-	0–0.6	Aida *et al*. [100]
EHA105	0.1 (660)	3	50	-	-	20	-	250	-	-	37	Shinoyama *et al*. [9]

OD: Optical density; CCP: co-culture period; K: kanamycin; VA: vancomycin; CF: cefotaxime; H: hygromycin; CA: carbenicillin; G: geneticin; TI: ticarcillin; P: paromomycin.

### Modeling procedures

Three individual machine learning algorithms including Multi-Layer Perceptron (MLP), Adaptive Neuro-Fuzzy Inference System (ANFIS), and Radial Basis Function (RBF) were proposed as estimator tools for modeling and optimizing chrysanthemum gene transformation datasets. The input variables were *Agrobacterium* strain, OD, CCP, and different antibiotics including K, VA, CF, H, CA, G, TI, and P. Also, the efficiency of gene transformation was chosen as outputs. Databases were randomly divided into three datasets: training set (70% database), testing set (20% database), and validation set (10% database). The MLP as one of the well-know ANNs was employed according to Hesami *et al*. [45] procedure. Also RBF and ANFIS were employed according to Hesami *et al*. [10] and Hesami *et al*. [13] procedures.

### Ensemble model

Ensemble is known as the process of combining and mixing data from various sources such as single outputs of several machine learning algorithms that the overall equation can be as follows;

$${\overset{\wedge }{y}}_{i}=f\left(x_{i}\right)+\varepsilon_{i}\;i=1,\;2,\;3,\ldots ,n$$ (1)

Where ${\overset{\wedge }{y}}_{i}$ stands for target variable, x is a vector of independent estimators, ε stands for corresponding estimation error, and n is a number of observation data.

In order to develop ensemble models, Eq (1) can be introduced to the following form where several individual models are employed;

$$\left[{\overset{\wedge }{y}}_{i}\right]=\left[\begin{array}{l} {\overset{\wedge }{y}}_{i1}\\ {\overset{\wedge }{y}}_{i2}\\ .\\ .\\ .\\ {\overset{\wedge }{y}}_{im} \end{array}\right]=\left[\begin{array}{l} f_{1}\;(x_{i})\\ f_{2}\;(x_{i})\\ .\\ .\\ .\\ f_{m}\;(x_{i}) \end{array}\right]+\left[\begin{array}{l} \varepsilon_{i1}\\ \varepsilon_{i2}\\ .\\ .\\ .\\ \varepsilon_{im} \end{array}\right]\;i=1,2,\ldots ,n$$ (2)

Where m stands for the number of individual model and [${\overset{\wedge }{y}}_{i}$] stands as matrix of estimations provided by each model.

Subsequently, the matrix of [${\overset{\wedge }{y}}_{i}$] will be considered as input data infusion models.

**Fig 2:**
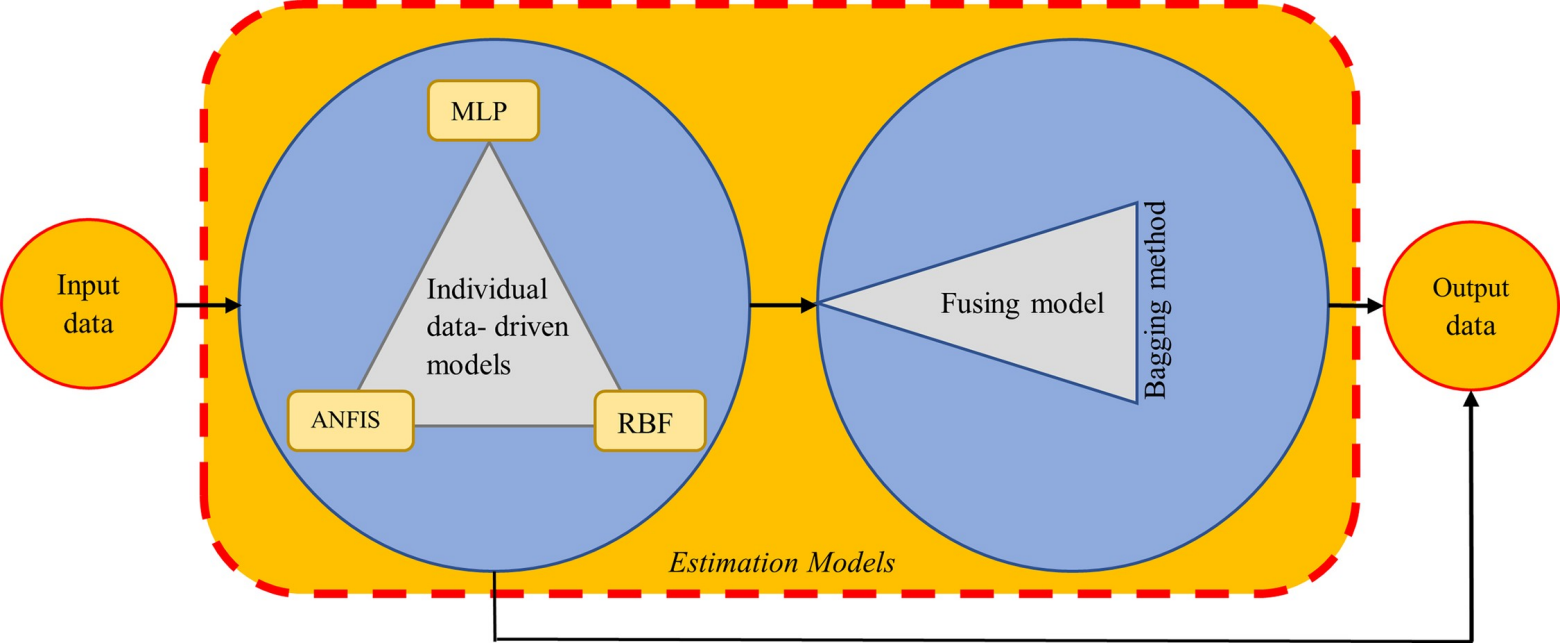
The schematic view of the proposed ensemble model.

Many methods have been recommended for fusing individual models, which reported that the most powerful and uncomplicated among different approaches is the bagging method for data fusing. Therefore, the best-resulted outputs achieved by three individual models were fused through the bagging method (Fig 2).

Finally, the coefficient of determination (R^2^), Mean Bias Error (MBE), and Root Mean Square Error (RMSE) were employed to determine the predictive ability of the developed model.

### Fruit fly optimization algorithm (FOA)

**Fig 3:**
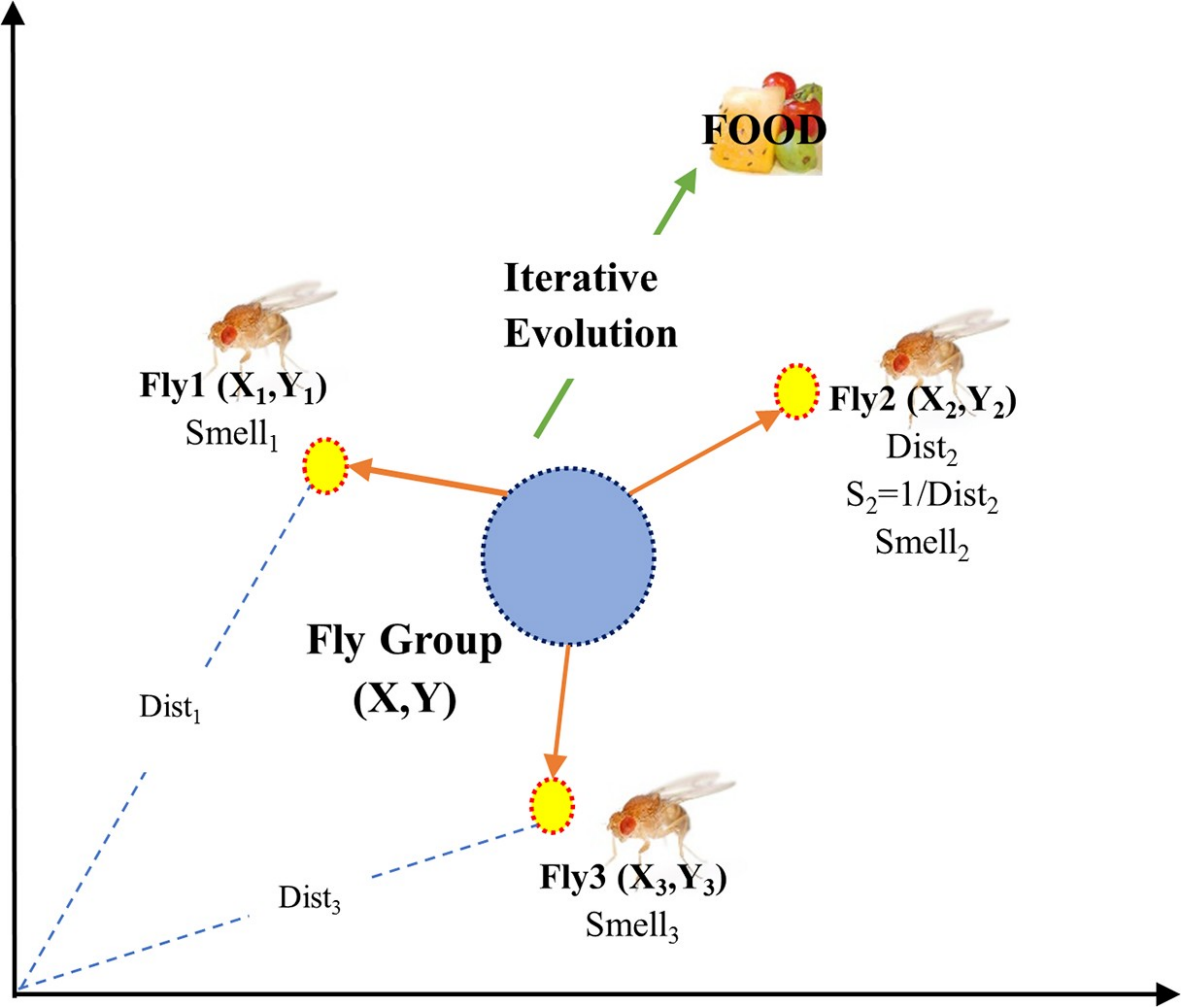
The schematic view of the fruit fly optimization algorithm (FOA).

The FOA is a novel approach for selecting optimization based on the food-finding activities of the fruit fly (Fig 3). The fruit fly is a type of insect, which lives in the tropical and temperate regions and eats corrupt fruit. In the current study, the FOA was applied to find optimal levels of inputs for achieving the maximum gene transformation efficiency. The details of the FOA are presented as follows:

#### Step 1: Initialization parameters

First, the maximum repeat number (*maxgen*), the initial fruit fly swarm location (*X_axis*,*Y_axis*), the population size (*sizepop*), and the random flight distance range (*FR*) should be considered. In this investigation, maxgen = 100, (X_axis, Y_axis) ⸦ [0,1], sizepop = 10, and FR ⸦ [–10,10] were considered.

#### Step 2: Evolution starting

The generation = 0, and the random flight path and the route for food finding of a single fruit fly were considered.

#### Step 3: Preliminary computations

The flight distance (*Dist*_*i*_) of food finding of the fruit fly *i* were adjusted. Subsequently, the smell concentration decision value *Si* were determined. *Si* were entered into the GRNN model. Then, the fitness function value (also called the smell concentration *Smell*_*i*_) was assessed. The fitness function value was used as the root-mean-square error (RMSE) which calculates the deviation between the actual value and the forecasting value.

#### Step 4: Offspring generation

The offspring generation is produced according to the following Equations:

$$X_{i}=X‐axis+Random\;Value$$ (3)

$$Y_{i}=Y‐axis+Random\;Value$$ (4)

$$Dist_{i}={\left(X_{i}{}^{2}+Y_{i}{}^{2}\right)}^{1/2}$$ (5)

$$S_{i}=1/Dist_{i}$$ (6)

$$Smell_{i}=Function\;\left(S_{i}\right)$$ (7)

$$\left[bestSmell\;bestIndex\right]=max\;\left(Smell_{i}\right)$$ (8)

Smellbest=bestSmell (9)

X‐axis=X(bestIndex) (10)

Y‐axis=Y(bestIndex) (11)

Then the offspring was linked to the ensemble model and the fitness function value again was determined. Also, generation = generation + 1 was considered.

#### Step 5: Circulation stops

When the generation attains the maximum repeat number, the stop criterion would be satisfied, and the optimized parameter value of the ensemble model can be reached. Otherwise, the optimization process should go back to Step 2.

### Sensitivity analysis

Sensitivity analysis was conducted to identify the importance degree of input variables on the efficiency of gene transformation. The sensitivity of these parameters was measured by the criteria including variable sensitivity error (VSE) value displaying the performance (RMSE) of the ensemble model when that input variable is removed from the model. Variable sensitivity ratio (VSR) value was determined as ratio of VSE and ensemble model error (RMSE value) when all input variables are available. A higher important variable in the model was detected by higher VSR.

MATLAB (Matlab, 2010) software was employed to write codes and run the models.
